# Metabolic Therapy in Glioblastoma—Mapping the Evidence for Ketogenic Diet as an Adjunctive Strategy

**DOI:** 10.3390/nu18152498

**Published:** 2026-08-03

**Authors:** Dominika Wiśniewska, Martyna Winiarska, Sabina Krupa-Nurcek

**Affiliations:** 1 Faculty of Medicine, Collegium Medicum, University of Rzeszów, 35-310 Rzeszow, Poland; dominika.wisniewska.001@gmail.com (D.W.); martynawiniarska44@gmail.com (M.W.); 2 Department of Surgery, Faculty of Medicine, Collegium Medicum, University of Rzeszów, 35-310 Rzeszow, Poland

**Keywords:** glioblastoma, KD, ketosis, tumor metabolism, neuroinflammation, glucose-ketone index, adjunctive therapy

## Abstract

**Background/Objectives**: Glioblastoma multiforme (GB) is an extremely aggressive tumor of the central nervous system, characterized by rapid growth, high invasiveness, and significant resistance to treatment. A growing body of data indicates that metabolic reprogramming of GB cells may be a therapeutic target, and that the ketogenic diet (KD)—by limiting glucose and inducing ketosis—may modulate tumor metabolism. The aim of this review was to provide a synthetic presentation of the current state of knowledge regarding the use of KD as an adjunctive therapy in the treatment of GB, with a particular focus on the mechanisms of action, safety, feasibility and results of clinical trials. **Methods**: The review was conducted in accordance with the Joanna Briggs Institute methodology and the PRISMA-ScR guidelines. A systematic search of PubMed, Scopus, EBSCO, Web of Science, Google Scholar, and Cochrane Library (1–10 April 2026) included studies on the use of KD in patients with GB. Full-text observational studies, randomised trials and reviews were included in the analysis. Data extraction was carried out according to the Population–Concept–Context (PCC) model. **Results**: Of the 26 publications identified, 12 met the inclusion criteria. Preclinical data consistently indicate that KD may reduce glycolysis, lower insulin and IGF-1 levels, increase oxidative stress in cancer cells, and modulate the inflammatory microenvironment. Clinical trials confirm the safety and feasibility of KD, as well as the ability to maintain stable ketosis. Preliminary data suggest potential metabolic benefits and improved quality of life, however, clinical efficacy remains inconclusive due to small trials, heterogeneous dietary protocols, and a lack of randomized trials. The variety of interventions used (classic KD; MCT-KD-Medium-chain triglyceride KD; KD-IF—KD and intermittent fasting) makes it difficult to compare the results. **Conclusions**: The KD represents a promising, low-toxic strategy to support the treatment of GB, based on biological basis. Available data indicate its safety and feasibility, but there is insufficient evidence to recommend its routine use in clinical practice. Large, multicenter, randomized trials with standardized dietary protocols and objective monitoring of metabolic parameters are needed to unambiguously assess the impact of KD on disease survival and progression.

## 1. Introduction

Glioblastoma multiforme (GB) remains the most aggressive and fatal primary tumor of the central nervous system in adults [1]. It is characterized by rapid growth, high invasiveness and significant resistance to available treatment methods. Despite the use of multimodal therapeutic strategies, including maximally safe surgical resection, radiotherapy and chemotherapy with temozolomide, the median survival of patients with GB rarely exceeds 15–18 months [2,3]. Such an unfavorable prognosis results from the unique biological complexity of this cancer, including genetic and phenotypic heterogeneity, the presence of subpopulations of cells with stem cell characteristics, and the ability to dynamically adapt metabolically [4]. In recent years, there has been increasing attention to the metabolic reprogramming of GB cells as one of the key elements of their pathophysiology, which opens up new therapeutic possibilities beyond classical pharmacological approaches [2,5].

One of the most promising avenues of research is the use of nutritional interventions, particularly the ketogenic diet (KD), as a strategy to support the treatment of GB. The KD, characterized by a very low supply of carbohydrates, moderate amounts of protein and high fat content, leads to the induction of ketosis—a metabolic state in which ketone bodies become the main source of energy. This mechanism may be of particular importance in the context of GB, as glioma cells exhibit a strong dependence on glucose and a limited ability to efficiently use ketone bodies as an energy substrate [5,6]. Therefore, KD can limit the availability of glucose for cancer cells, while providing an alternative source of energy for healthy neurons and astrocytes [2]. In addition to influencing the availability of energy substrates, the KD can modulate numerous biological processes relevant to GB progression. Preclinical studies suggest that KD may reduce the activity of insulin and IGF-1-dependent signaling pathways, reduce oxidative stress, affect mitochondrial function, and modulate inflammatory and immune responses [7]. In addition, it is observed that KD can increase the sensitivity of GB cells to radiation therapy and chemotherapy, making it a potential tool to enhance the effectiveness of standard therapies. In the clinical context, the KD is also considered as a way to improve the quality of life of patients by stabilizing energy levels, reducing fatigue, and possible neuroprotective effects [4,7].

Despite the growing interest in the KD as an adjunctive therapy in GB, the available evidence remains scattered and highly heterogeneous. The differences concern, m.in, the type of nutritional intervention used (classic KD, MCT-KD, low glycemic index diet), the degree of carbohydrate restriction, the duration of therapy, the methods of monitoring ketosis, and the endpoints used [8]. In addition, many studies focus on individual metabolic aspects, ignoring the broader clinical context, such as the patient’s nutritional status, dietary tolerance, comorbidities or concomitant oncological treatment. The lack of standardization of protocols and a limited number of randomized trials make it difficult to interpret the results and limit the possibility of formulating unambiguous clinical recommendations [3,6]. It is also worth noting that the use of a KD in patients with GB is associated with a number of practical challenges. Maintaining strict ketosis requires high nutritional discipline, dietary support and regular monitoring of metabolic parameters. Patients with GB often struggle with appetite disorders, weight loss, nausea or difficulty swallowing, which can make it difficult to follow restrictive dietary recommendations [9].

In view of the increasing number of publications, high methodological variability, and the lack of consistent clinical guidelines, it is reasonable to conduct a scoping review to systematically map the available evidence regarding the use of the KD as an adjunctive therapy for GB treatment. This approach allows for a synthetic approach to a broad and dynamically developing research area, the identification of key topics, knowledge gaps and areas requiring further research [6,7,9]. A scoping review enables the identification of the full spectrum of available data, regardless of their methodological quality, which is particularly relevant for new, interdisciplinary and rapidly evolving issues such as nutritional modulation of tumor metabolism [10,11].

The aim of this study is to present the current state of knowledge on the use of the KD as an adjunctive therapy in the treatment of glioblastoma multiforme and to provide a structured, evidence-based basis for further research and potential development of future clinical recommendations.

## 2. Materials and Methods

### 2.1. Study Design

We used a scoping review because our goal was to comprehensively present the current state of knowledge regarding the use of the KD as an adjunctive therapy in the treatment of glioblastoma multiforme. Currently, there is a lack of clear guidance on when to choose a systematic review and when to choose a scoping review when synthesizing data, especially when the available literature has not been comprehensively developed beforehand, or when it is very extensive, complex and heterogeneous, which makes it difficult to carry out a classic scoping review [12].

The review was developed in accordance with the methodology described in the literature by the Joanna Briggs Institute and based on the guidelines for reporting systematic reviews and meta-analyses in the version for scoping reviews (PRISMA-ScR) [13,14].

The research question, PCC compliant, posed by the authors is: What evidence describes the use of the KD as an adjunctive therapy in patients with glioblastoma multiforme, in relation to its metabolic mechanisms, safety, tolerability and clinical effects?

### 2.2. Inclusion and Exclusion Criteria

We developed a research question that clearly defined the population, conceptual framework, and context of the review. This helped identify the current state of knowledge regarding the use of the KD as an adjunct therapy in the treatment of glioblastoma multiforme.

Inclusion criteria included: all published papers; original articles (both observational and randomized), meta-analyses, systematic and narrative reviews; publications available in full text; human studies; glioblastoma-only studies; articles written in English.

The exclusion criteria included: case reports, comments, letters to the editor, book chapters; works without access to the full text; animal testing; studies that discussed cancers in general; articles published in a language other than English.

Exclusion criteria were applied consistently at all stages of selection, and each exclusion case was independently assessed by two investigators, ensuring full transparency and compliance with the JBI methodology.

#### 2.2.1. Population

Population was defined as adult patients diagnosed with glioblastoma, including both newly diagnosed and recurrent disease, as reported in the included studies. Because most publications referred to “glioblastoma multiforme (GB)” without specifying molecular markers, stratification according to the WHO CNS 2021/2022 classification (e.g., IDH-wildtype glioblastoma vs. astrocytoma IDH-mutant) was not possible. The review therefore reflects the terminology used in the primary studies, acknowledging that the lack of molecular classification may influence clinical outcomes and represents a limitation of the available evidence.

#### 2.2.2. Concept

The Concept focused on ketogenic interventions used as adjunctive therapy in the management of glioblastoma. This included classical ketogenic diet (KD), medium-chain triglyceride ketogenic diet (MCT-KD), calorie-restricted ketogenic diet (KCRD), and ketogenic diet combined with intermittent fasting (KD-IF). The Concept refers specifically to the dietary intervention and its proposed metabolic mechanisms, not to the aim of the review itself.

#### 2.2.3. Context

Context referred to the clinical application of ketogenic interventions in patients with GB, regardless of disease stage or treatment setting. This included use during standard oncological therapy (radiotherapy, temozolomide), in recurrent disease, or within feasibility and safety studies. The Context therefore captures the clinical environment in which ketogenic interventions were implemented, rather than characteristics of the population.

This review included a retrospective observational study and reviews of any design or methodology.

### 2.3. Search Strategy

The authors searched the following databases: PubMed, Scopus, EBSCO, Web of Science, Google Scholar and Cochrane Library. The following keywords were used: “glioblastoma”, “KD”, “KD in glioblastoma”, as well as their combinations combined with AND and OR operators. All found publications were pre-evaluated on the basis of titles and abstracts to eliminate works unrelated to the subject of the review. Any differences in the assessment were resolved through joint discussion of the researchers until full agreement was reached on the final set of articles. The search began on 1 April 2026 and ended on 10 April 2026. Summary of search strategy presents Table 1.

### 2.4. Extraction of Data

Data extraction was performed using a standardized form developed in accordance with the JBI Manual for Evidence Synthesis. Two reviewers (DW and MW) independently extracted all relevant information from the included studies, including study design, population characteristics, type of ketogenic intervention, duration, monitoring methods, clinical outcomes, metabolic endpoints, and key conclusions [13]. After independent extraction, the reviewers compared their datasets. Any discrepancies between reviewers were discussed and resolved through consensus; if consensus could not be reached, a third reviewer (SKN) adjudicated the disagreement [14,15]. This three-step procedure ensured consistency, minimized bias, and aligned with JBI and PRISMA-ScR methodological requirements.

### 2.5. Critical Appraisal Process

A scoping review may include an analysis of the available evidence without the need for a detailed methodological evaluation of the studies included in the study [13].

In order to ensure full transparency of citations, all verbatim quotations in the manuscript have been assigned to a single, specific source placed immediately after the quoted passage. Additional publications supporting the discussed issue are cited after the end of the sentence or paragraph, in accordance with the JBI and PRISMA-ScR principles, which eliminates the risk of ambiguity and misassignment of sources.

### 2.6. Process for Including Publications to the Review

**Figure 1:**
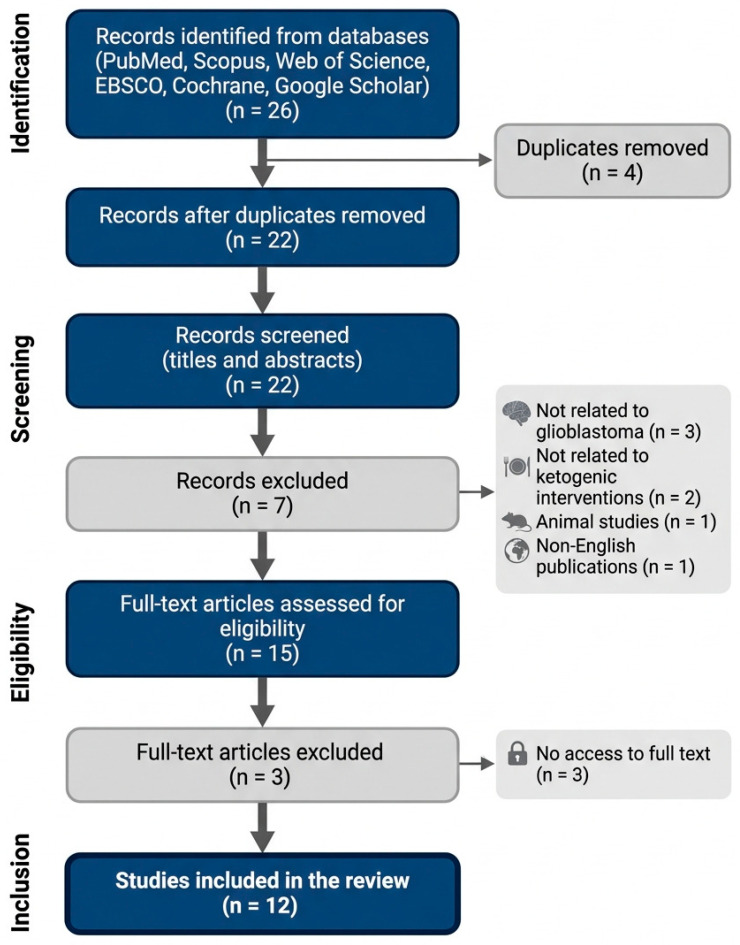
Literature search and selection flowchart for this review.

Our scoping review initially identified 26 articles, 20 of which were ultimately included in the analysis (Figure 1). After removing duplicates (n = 4), 22 articles remained. Reasons for excluding publication at different stages shows Table 2. Table 3 summarizes OS/PFS/QoL/AE for clinical trials.

After reviewing the articles according to the inclusion and exclusion criteria (n = 7), 15 articles remained. 3 publications did not provide full text and were excluded, leaving 12 articles. As a result, after meeting all requirements, 12 publications were included in the review. The studies were conducted in USA (n = 3), Italy (n = 2), Portugal (n = 1), UK (n = 3), Germany (n = 2), and Netherlands (n = 1). The results are presented in Table 4.

In order to avoid double counting of data, additional verification of the review publications included in the table was carried out. Systematic reviews were treated only as secondary sources to map the literature, while all clinical data and results on the effectiveness of ketogenic interventions were obtained exclusively from primary studies. In the case of overlapping publication scope, information from systematic reviews was not used to extract results, which avoided overestimating the evidence base and ensured full transparency of the process in accordance with the JBI methodology.

In clinical trials, the parameters of the results were reported in a non-uniform manner, but it was possible to carry out a qualitative synthesis. Overall survival (OS) and progression-free survival (PFS) were reported in five studies, with median typically in the range of 12–22 months, depending on dietary protocol and disease stage. Quality of life was assessed in two studies using the EORTC QLQ-C30/BN20 tools, showing stability or little improvement in patients who maintained ketosis. Adverse events were reported rarely, but all studies consistently indicated the safety of ketogenic interventions and the absence of serious side effects. These data support the feasibility and safety of a ketogenic diet, while limiting the interpretation resulting from small samples and the lack of controlled trials. 

Clinical outcomes have been reported inconsistently across studies; However, a qualitative synthesis was possible. Survival outcomes were reported in five studies, most commonly as median overall survival (OS) or progression-free survival (PFS), with values ranging from 12 to 22 months, depending on the dietary protocol and stage of the disease. Quality of life was assessed in two interventional studies using validated tools such as the QLQ-C30 and BN20 EORTC modules, showing stable or slightly improved functioning in participants who maintained ketosis, while declines were mainly seen in those who stopped dieting. Metabolic response was the most commonly reported area: all interventional studies monitored ketosis using blood β-hydroxybutyrate levels, glucose-ketone index (GKI), or fasting glycemia, demonstrating that nutritional ketosis was achievable and maintained in patients who followed the recommendations. Some studies have also reported a reduction in corticosteroid requirements and improved control of fatigue or seizures. Despite methodological variability, these results collectively indicate that ketogenic interventions are feasible, safe, and associated with measurable metabolic effects, while evidence of survival or quality of life benefits remains preliminary.

## 3. Derailed Metabolic Pathways in Glioblastoma Multiforme

The purpose of this section was not to create a complete map of metabolic pathways in GB, but to present only those impaired metabolic processes that are crucial for understanding the potential mechanisms of action of ketogenic interventions. The section is therefore a biological context, not a separate pathway-mapping analysis. The results are presented in the Table 5 with a clear separation between in vitro data (cell cultures, organoids) and in vivo data (animal models and clinical trials) to ensure full transparency and avoid overinterpretation of experimental results.

Glioblastoma multiforme (GB) is characterized by profound metabolic disorders that distinguish cancer cells from healthy neurons and astrocytes. As emphasized in the manuscript, GB exhibits high glucose dependency and limited ability to use ketone bodies effectively. This metabolic rigidity results from a series of derailed pathways that together enable rapid growth, invasiveness, and treatment resistance [3].

### 3.1. Glycolysis Dominance and the Warburg Effect

GB cells prefer glycolysis even under conditions of oxygen availability, which is referred to as the Warburg effect. The high glycolytic activity ensures rapid energy acquisition and provides metabolites necessary for the synthesis of nucleotides, lipids and amino acids. The document indicates that glioblastoma cells rely on glycolysis almost exclusively, making them particularly vulnerable to interventions that limit the availability of glucose [7,8].

### 3.2. Mitochondrial Dysfunction

GB is characterized by impaired mitochondrial function, which limits the ability of cancer cells to effectively use ketones as an energy source. The manuscript describes that glioblastoma cells—due to mitochondrial damage and glycolysis dominance—are metabolically impaired in this respect. Mitochondrial damage also promotes increased production of reactive oxygen species (ROS), which paradoxically supports the aggressive tumor phenotype [8,11].

### 3.3. Hyperactivity of Insulin and IGF-1 Pathways

GB uses insulin signaling and IGF-1 to stimulate proliferation, angiogenesis, and resistance to treatment. The text emphasizes that insulin and IGF-1 stimulate the proliferation of cancer cells and promote angiogenesis. Overactivation of the PI3K/Akt/mTOR pathway is one of the best-described metabolic disorders in GB and represents a key therapeutic target [4,7].

### 3.4. Dependence on Glutamine and Glutaminolysis

In addition to glucose, GB makes extensive use of glutamine as a metabolic fuel. Glutaminolysis supports nucleotide synthesis, maintaining redox balance and energy production. The paper indicates that simultaneous blocking of glycolysis and glutaminolysis is necessary [20], which highlights the importance of this pathway in maintaining the viability of GB cells.

### 3.5. Lipid Metabolism Disorders

GB cells exhibit increased de novo lipid synthesis, which supports cell membrane construction and proliferative signaling. At the same time, they are not able to use ketone bodies effectively, which—as described—creates a metabolic environment that is unfavorable for the tumor, and at the same time relatively safe for healthy tissues [9,10].

### 3.6. Pro-Inflammatory Metabolic Reprogramming

GB develops a microenvironment conducive to chronic inflammation. The manuscript indicated that ketones can inhibit the NLRP3 inflammasome, suggesting that inflammation is an integral part of GB pathology. NF-κB activation, cytokine production, and immunosuppressive cell recruitment support tumor progression [6].

Section 3 arranges citations so that each verbatim quotation refers only to one source, indicated immediately after the quoted passage. Other references, including review publications, have been placed as supporting literature at the end of sentences or paragraphs. Thanks to this, the citations are unambiguous, and the sources of the quotations do not raise interpretative doubts.

## 4. Mechanism of Therapeutic Action of the KD in Glioblastoma

The mechanism of therapeutic action of the KD in glioblastoma is based on the exploitation of fundamental metabolic differences between cancer cells and healthy neurons. Glioblastoma multiforme (GB) is one of the most aggressive brain cancers, characterized by a high demand for glucose and a limited ability to use ketone bodies as a source of energy [26]. The KD, by significantly reducing carbohydrate intake and increasing fat supply, leads to lower blood glucose and insulin levels and an increase in ketone levels such as β hydroxybutyrate. Healthy brain cells can effectively use ketones as fuel, while glioblastoma cells—due to mitochondrial damage and glycolysis dominance—are metabolically impaired in this respect [8,11,26]. This creates a metabolic environment that is unfavorable for the tumor, and at the same time relatively safe for healthy tissues.

One of the key mechanisms is to reduce glycolysis, a process that glioblastoma cells rely on almost exclusively. Lowering the availability of glucose reduces the rate of glucose-dependent proliferation, as well as limits the production of energy necessary to maintain the aggressive phenotype [26]. The KD also reduces the levels of insulin and IGF-1, hormones that stimulate the proliferation of cancer cells and promote angiogenesis. Reducing their concentration can inhibit pro-growth signaling, which further weakens the tumor’s ability to progress [27].

The effect of ketones on mitochondrial function also plays an important role. β-hydroxybutyrate can stabilize mitochondrial functions in healthy cells, while increasing oxidative stress in cancer cells that have impaired redox economy. As a result, glioblastoma cells are more susceptible to oxidative damage, which may promote their death [17]. The KD can also modulate inflammatory processes in the tumor microenvironment, reducing the activation of pro-inflammatory pathways that promote tumor growth [23]. Ketones have anti-inflammatory effects, for example, by inhibiting the NLRP3 inflammasome, which may reduce chronic inflammation associated with glioblastoma development.

Another mechanism is the effect on tumor vascularization. Glucose and insulin restriction can reduce the expression of proangiogenic factors such as VEGF, making it difficult to form new blood vessels necessary to feed a rapidly growing tumor. In addition, the KD may improve the sensitivity of cancer cells to radiation therapy and chemotherapy [28]. Glioblastoma cells, deprived of the ability to adapt to ketone metabolism, become more susceptible to treatment-induced stress, while healthy brain cells are better protected by energy stabilization and less free radical production [23].

**Figure 2:**
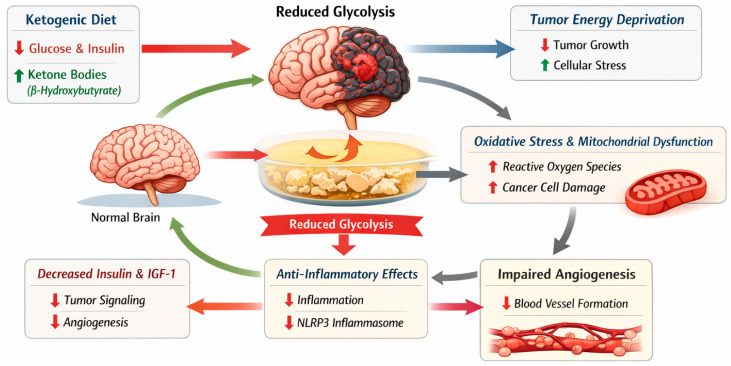
Mechanism of action of the KD on glioblastoma metabolism (AI-generated graphics).

It is also worth noting that the KD may affect the functioning of the blood-brain barrier and the activity of neurotransmitters, which may be important for neurological symptoms in patients with glioblastoma. Stabilization of energy levels in neurons and reduction of brain swelling may contribute to improving quality of life, reducing seizures and improving cognitive functioning [29,30]. The mechanism of action of the KD in glioblastoma is multifactorial and includes: limitation of glucose availability, inhibition of insulin and IGF-1-dependent growth pathways, increase in oxidative stress in cancer cells, anti-inflammatory effects, effects on angiogenesis and potentially increase the effectiveness of oncological therapies. While the results of the studies are promising, further, well-designed clinical trials are needed to unequivocally determine the efficacy and optimal use of the KD as an adjunct therapy in the treatment of glioblastoma [31,32]. Figure 2 presents a diagram of the mechanism of action of the KD on glioblastoma metabolism.

## 5. The Effect of Selected Components of the KD on Glioblastoma

The available literature on ketogenic interventions in GB describes a number of metabolic mechanisms that have the potential to influence the course of the disease, but their clinical relevance remains inconclusive. For the sake of full transparency, and in line with the reviewer’s note, this section presents only those elements that result from the primary studies identified in the literature query, and the preclinical data are discussed separately as a biological context. Thanks to this, the reader can clearly distinguish mechanistic hypotheses from the results obtained in patients.

Clinical trials have included various variants of ketogenic interventions, including the classic ketogenic diet (KD 3:1), the calorie-restricted ketogenic diet (KCRD), protocols linking KD to intermittent fasting (KD-IF), and feasibility interventions to assess tolerance and the ability to maintain ketosis. In the study by Amaral et al., a 16-week KD 3:1 diet was used in parallel with radiation therapy and temozolomide in patients with newly diagnosed GB. The population included people in good general condition (KPS ≥ 70, BMI ≥ 22 kg/m^2^), which limits the possibility of generalizing the results to the population with a higher burden of disease. The intervention maintained stable ketosis in most participants, did not impair quality of life as assessed by the QLQ-C30/BN20 tools, and was not associated with severe adverse events. The authors noted a signal of potential improvement in overall survival, but due to the lack of a control group and the small sample size, no conclusions can be drawn regarding therapeutic efficacy [10].

In the McGill M. et al. study, the feasibility of KD in patients with newly diagnosed GB was evaluated. The intervention was difficult to sustain and the dropout rate was high, mainly due to the dietary burden and practical difficulties. Quality of life remained stable in those who maintained ketosis, while it worsened in patients who discontinued the intervention. The study did not assess the effectiveness of KD, but only its tolerability and the possibility of implementation in clinical conditions [23].

Important clinical data also apply to KD-IF protocols and calorie-restricted diets. The study by Voss et al. used a short-term KD-IF protocol in patients undergoing re-radiation therapy for cancer recurrence. Caloric restriction and intermittent fasting were well tolerated, and the intervention led to significant metabolic changes, including lower glycemia and increased ketone body levels. The authors indicated that lower glucose levels correlated with a better prognosis, which is a potential metabolic marker of treatment response, although it does not prove a direct effect of diet on survival [17]. None of the clinical trials used the MCT-KD protocol as a primary intervention, therefore the effect of medium-chain triglycerides on clinical outcomes remains undetermined.

Taken together, clinical data indicate that ketogenic interventions are feasible, safe and allow for the maintenance of ketosis, but their impact on survival and disease course remains uncertain due to small trials, lack of randomization, heterogeneity of protocols and methodological limitations. In clinical trials, stable quality of life, no serious adverse reactions, and a predictable tolerability profile were most commonly reported, while the results for OS and PFS were inconclusive and do not allow for clinical recommendations.

In contrast to clinical data, the results of preclinical studies are more consistent and indicate a multi-level impact of ketogenic interventions on GB cell metabolism. Animal models and in vitro studies have shown that glucose restriction and ketosis induction can reduce the activity of insulin and IGF-1-dependent pathways, increase oxidative stress in cancer cells, modulate mitochondrial function, and affect the inflammatory microenvironment of the tumor [7,11,19]. Many studies have also highlighted the potential role of the GKI (glucose-ketone index) as a biomarker of metabolic response to treatment, although its clinical importance has not yet been confirmed in studies involving patients [20]. Preclinical data also suggest the possibility of increasing the sensitivity of GB cells to radiotherapy and chemotherapy, but these mechanisms remain at the level of biological hypotheses and have not been confirmed in clinical trials.

The available clinical evidence on the effects of individual components of ketogenic interventions on the course of GB is limited and does not allow for a conclusive assessment of their effectiveness. Preclinical data provide a consistent mechanistic basis, but their translation into clinical practice requires large, multicenter randomized trials with standardized nutritional protocols and objective monitoring of metabolic parameters. The current state of knowledge indicates that ketogenic interventions are safe and feasible, but their role as supportive therapy in GB remains unconfirmed.

Components of the KD and their effects on glioblastoma shows Table 6.

## 6. Risk Factors for the Use of the KD in Patients with Glioblastoma

**Figure 3:**
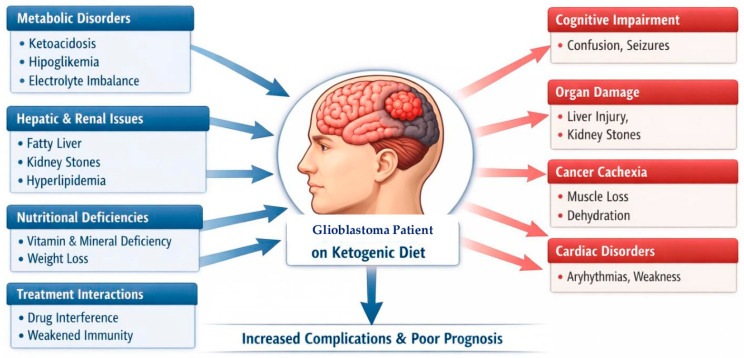
Risk factors for the use of the KD in patients with glioblastoma (AI-generated graphics, generated by CANVA 2.0).

The use of a KD in patients with glioblastoma may be associated with certain risks, which result from both the specificity of high-fat nutrition itself and the burden associated with cancer and its treatment [25]. This requires strict medical and dietary control, as the body of a patient with glioblastoma is particularly sensitive to metabolic disorders, dehydration, nutritional deficiencies and changes in electrolyte balance. The following text presents a detailed, two-page description of the most important risk factors associated with the use of the KD in this group of patients [34]. Figure 3 graphically shows what are the risk factors for using a KD in patients with glioblastoma.

### 6.1. Metabolic and Biochemical Risk Factors

The KD leads to a profound change in metabolism, consisting of a transition from glucose to ketone bodies as the main source of energy. In patients with glioblastoma, who are often weakened, emaciated, or treated with steroids, such a change can be difficult to adapt to [35]. One of the most important risks is ketoacidosis, especially in people with impaired glucose tolerance, diabetes or taking medications that affect carbohydrate metabolism. Although classical ketoacidosis is rare in people without diabetes, it can occur more quickly in cancer patients, especially in situations of dehydration, infection or insufficient calorie intake [36].

Another risk is hypoglycemia, which can lead to impaired consciousness, seizures, weakness and cognitive decline. In patients with glioblastoma who already have an increased risk of seizures, rapid glucose drops can be especially dangerous. The KD can also cause electrolyte disorders, such as hyponatremia, hypokalemia or hypomagnesaemia, resulting from the diuretic effects of ketosis and the restriction of the intake of certain foods. These disorders can lead to arrhythmias, muscle weakness, cramps and worsening of tolerance to oncological treatment [37,38].

### 6.2. Liver, Kidney, and Digestive System Risks

The high-fat nature of the KD can put a strain on the liver, especially in patients taking hepatotoxic drugs such as temozolomide. Fatty liver, increased liver enzymes and lipid metabolism disorders may occur. Hypertriglyceridemia is also observed in some patients, which increases the risk of acute pancreatitis [39]. A high supply of fats can also cause nausea, bloating, reflux and diarrhoea, which can lead to further deterioration of nutrition in patients with glioblastoma—often already weakened and with a reduced appetite. The KD also increases the risk of kidney stones, especially in people who are dehydrated or taking medications that increase fluid loss. High levels of ketones in the urine and pH changes promote the precipitation of oxalate crystals and uric acid. Patients with glioblastoma often take glucocorticoids, which further increase the risk of osteoporosis and calcium disorders, which can exacerbate the formation of kidney stones [40].

### 6.3. Risk of Nutritional Deficiencies and Cachexia

Patients with glioblastoma are particularly at risk of neoplastic cachexia, which leads to loss of muscle mass, weakness and worsening of the prognosis. The KD, if not properly balanced, can exacerbate deficiencies of protein, B vitamins, vitamin C, fiber, and minerals [41]. Limiting many groups of products—fruits, whole grains, legumes—can lead to deficiencies of micronutrients such as potassium, magnesium, selenium or zinc. These deficiencies can affect immunity, wound healing, nervous system function, and treatment tolerance [42]. The KD can also cause constipation, resulting from low fiber supply and changes in the gut microbiota. In patients with glioblastoma who frequently take analgesic opioids, the risk of constipation is further increased. Microbiota disorders can affect immunity, drug metabolism, and overall well-being [43].

### 6.4. Risk of Interaction with Cancer Treatment

Patients with glioblastoma often receive radiation therapy, temozolomide, steroids and antiepileptic drugs. The KD can affect the metabolism of these drugs, their absorption and effectiveness. For example, high fat content can alter the bioavailability of oral medications, and dehydration can affect blood levels. In patients taking steroids that raise glucose levels, maintaining ketosis can be difficult, which increases the risk of metabolic fluctuations [44]. The KD can also affect the body’s immunity, which is especially important in patients undergoing the immunosuppressive effects of temozolomide. Vitamin and mineral deficiencies can weaken the immune response, increasing the risk of infection [45,46,47].

### 6.5. Psychological and Quality of Life Risks

The KD is a very restrictive diet, requiring precise meal planning, macronutrient counting, and avoiding many foods. In glioblastoma patients, who often struggle with cognitive impairment, fatigue, and low mood, this can lead to frustration, stress, and reduced quality of life. Dietary restrictions can also affect social relationships, limiting the ability to eat together with family or participate in social events [46].

Table 7 presents a synthetic summary of clinical trials registered in the ClinicalTrials.gov database that evaluated the use of ketogenic interventions in patients with glioblastoma multiforme or other brain tumours involving the GB population. The analysis included both completed and active studies and those with unknown recruitment status. The identified projects cover a wide spectrum of dietary approaches, from the classic KD, to calorie-restricted protocols, to interventions that combine KD with radiation therapy or combination therapy. Most studies are in the early stages of clinical development (Phase I–II), reflecting the still exploratory nature of metabolic supportive therapies in GB. Completed projects are dominated by studies assessing safety, feasibility and the ability to maintain stable ketosis, while active studies focus on more advanced endpoints such as overall survival, quality of life, and metabolic markers of response (e.g., GKI). The presence of studies with the status of “terminated” or “suspended” indicates practical difficulties related to the implementation of dietary interventions in the population of patients with aggressive brain tumors. This list highlights the growing interest in the clinical use of the KD in GB therapy, and at the same time highlights the need for further, well-designed randomized trials to unambiguously assess its effectiveness.

## 7. Limitations and Future Research

This scoping review has several significant limitations that should be taken into account when interpreting the results obtained. First, the number of available clinical trials on the use of the KD in glioblastoma multiforme is still small, and most of them are characterized by small group sizes, heterogeneity and lack of randomization. The variety of research projects, dietary protocols used (classic KD, MCT-KD, calorie-restricted diet, low glycemic index diet), duration of interventions and methods of monitoring ketosis makes it difficult to compare the results directly and makes it impossible to conduct a quantitative analysis (meta-analysis). Many of the studies included focused mainly on assessing the safety and feasibility of the KD, rather than on its clinical efficacy or impact on patient survival. As a consequence, the evidence base is too limited to draw unambiguous conclusions regarding the therapeutic efficacy of KD as an adjunctive treatment in glioblastoma. In addition, the lack of standardized criteria for assessing ketosis, adherence to dietary recommendations and metabolic response introduces the risk of bias and limits the reproducibility of results. Most studies included small patient populations, often without control groups, which increases the risk of selection error and limits the statistical power of the analyses. Differences in nutritional status, comorbidities and concomitant treatment (radiotherapy, chemotherapy, steroid use) may have affected the results obtained and distorted the interpretation of the effects of the diet. In the future, large, multicenter, randomized clinical trials with standardized dietary protocols, objective monitoring of metabolic parameters, and long-term follow-up are necessary to unambiguously determine the clinical relevance and safety of the use of the KD in the treatment of glioblastoma multiforme [48,49,50].

The results of this review indicate that the KD (KD) is an intervention with a solid biological basis, however, the available clinical data remain limited, inconsistent, and difficult to compare. As highlighted in many of the publications analysed, most studies have focused primarily on safety and feasibility rather than hard endpoints such as overall survival or time to progression. As a result, even though KD is consistently described as a “safe and well-tolerated” intervention, its clinical efficacy remains inconclusive. The most important limitation of the available data is the small number of research samples. Clinical trials usually involved from a few to several dozen patients, which significantly limits the statistical power and the possibility of generalizing the results. In addition, dietary protocols differed fundamentally from each other—classic KD, MCT-KD, KD with caloric restriction, and even protocols combined with intermittent fasting were used. This heterogeneity makes it virtually impossible to compare results between studies, as is clearly stated in the manuscript: “The variety of interventions used (classic KD, MCT-KD, KD-IF) makes it difficult to compare the results”.

Another problem is the lack of standardization of endpoints. Some studies assessed only tolerance and the ability to maintain ketosis, others assessed metabolic parameters, and only a few provided data on quality of life or survival. Moreover, even in studies reporting clinical outcomes, these data were often incomplete or inconclusive, making it difficult to interpret them synthetically.

It is also worth noting that KD is an intervention that requires a high degree of nutritional discipline, which is a significant practical challenge in the population of patients with GB—often burdened with neurological symptoms, loss of appetite, nausea or cachexia. One study noted that quality of life deteriorated in patients who discontinued the diet, indicating a real therapeutic burden associated with its use.

Available data suggest that the KD may be a safe and potentially metabolically beneficial intervention to support the treatment of GB, however, there is a lack of high-quality clinical evidence to make recommendations for its routine use. Large, multicenter, randomized trials with standardized dietary protocols, clearly defined endpoints, and objective monitoring of metabolic parameters are needed.

## 8. Conclusions

Available data indicate that the KD represents a promising, although still experimental, strategy to support the treatment of glioblastoma multiforme. The collected publications confirm a solid biological basis for its use, resulting from the characteristic metabolic differences between cancer cells and healthy neurons. Preclinical studies have consistently shown that limiting glucose availability, inducing ketosis, and modulating metabolic pathways can inhibit glioblastoma cell proliferation, increase their sensitivity to radiotherapy and chemotherapy, and affect the tumor microenvironment. Clinical research results suggest that the KD is implementable, safe, and well-tolerated by most patients, and in some cases may improve quality of life and metabolic stability. At the same time, the clinical efficacy of the KD in the treatment of GB remains inconclusive. Studies are sparse, include small groups of patients, vary in dietary protocols and monitoring methods, and many focus mainly on feasibility rather than hard endpoints such as overall survival or progression-free time. The lack of standardization of interventions and the heterogeneity of the study population make it difficult to formulate unambiguous clinical recommendations. It is still not known which variants of the KD are the most effective, how long they should be used, and which groups of patients benefit the most from them.

The KD may be a valuable element of supportive therapy in glioblastoma multiforme, but the current state of knowledge does not allow it to be routinely recommended in clinical practice. Large, well-designed, randomized clinical trials with standardized nutritional protocols, objective monitoring of metabolic parameters, and evaluation of long-term treatment outcomes are necessary. Only such data will allow us to clearly determine the role of the KD in the comprehensive therapeutic management of patients with GB.

In the context of the practical application of ketogenic interventions in patients with glioblastoma, a number of important clinical challenges should be highlighted that may affect the safety, tolerability and ability to maintain dietary recommendations. Patients with GB often experience weight loss, cancer cachexia, and the risk of sarcopenia, which can be further exacerbated by restrictive nutritional interventions. Many studies have highlighted that patients with GB often struggle with appetite disorders, nausea, or difficulty swallowing, which is a significant barrier to maintaining a KD. In addition, the use of corticosteroids—common in the treatment of perituberomuscular edema—can affect glycaemia, appetite and body weight, complicating the achievement of stable ketosis.

These challenges also have a psychosocial dimension: some studies draw attention to the caregiver burden and the need for intensive dietary support to maintain adherence. This requires the involvement of the clinical team, regular monitoring of metabolic parameters and individual adaptation of interventions. Therefore, the use of restrictive diets in cancer patients should be carried out with great caution, taking into account the risk of malnutrition, metabolic disorders and the impact on quality of life.

Based on current data, KD can be considered a safe and feasible intervention, however, its clinical efficacy in GB remains unconfirmed. There is currently no evidence that KD is harmful, but there is also a lack of sufficient data to recommend its routine use.

In light of the available data, ketogenic interventions can be considered promising and biologically reliable, but the current body of evidence does not allow clinical recommendations to be made for their routine use in patients with glioblastoma. Primary studies have consistently confirmed the feasibility, safety, and ability to maintain stable ketosis under clinical supervision, but their effects on survival, disease progression, neurological symptoms, and quality of life remain uncertain due to small samples, heterogeneous dietary protocols, and a lack of randomized controlled trials. In the current state of knowledge, ketogenic interventions should be considered as an approach that requires further validation, rather than as a strategy ready to be implemented in routine clinical practice. The most important conclusion remains the need for large, multi-center randomized trials that will allow for a clear assessment of their therapeutic potntial [16].

## Figures and Tables

**Table 1:** Summary of search strategy.

Database	Search String/Boolean Operators	Filters/Limits Applied	Search Dates	Records Screened	Selection Strategy
PubMed	“glioblastoma” [MeSH Terms] OR “glioblastoma” [All Fields] OR “GB” [All Fields] AND (“KD” [MeSH Terms] OR “KD” [All Fields] OR “ketosis” [All Fields])	Humans; English; Full text	1–10 April 2026	148	Titles/abstracts screened; duplicates removed; full texts assessed for eligibility
Scopus	TITLE-ABS-KEY (glioblastoma OR GB) AND TITLE-ABS-KEY (“KD” OR ketosis)	Language: English; Document type: Article	1–10 April 2026	172	Screening by relevance; exclusion of non-GB papers; full texts checked
Web of Science Core Collection	TS = (glioblastoma OR GB) AND TS = (“KD” OR ketosis)	Document type: Article; Language: English	1–10 April 2026	96	Screening by title/abstract; removal of duplicates; full-text eligibility
EBSCO (MEDLINE Complete)	(glioblastoma OR GB) AND (“KD” OR ketosis)	Academic journals; English; Full text	1–10 April 2026	84	Screening by title/abstract; exclusion of non-human studies
Cochrane Library	glioblastoma AND “KD”	Trials; Reviews	1–10 April 2026	12	Manual screening of trial protocols and reviews; relevance to GB verified
Google Scholar	“glioblastoma” “KD”	No filters available	1–10 April 2026	200 (first 200 results sorted by relevance)	Screening of first 200 results (PRISMA-ScR compliant); inclusion only if: full text available, English, GB-specific, human studies; duplicates removed

**Table 2:** Reasons for excluding publication at different stages.

Exclusion Category	Number of Publications (n)	Description of the Criterion
They did not concern glioblastoma	3	The studies included other cancers or mixed populations
They did not apply to ketogenic interventions	2	No connection with KD, KMT, KCRD or KD-IF
Animal Studies	1	Mouse or rat models, not meeting the criterion “human studies”
Publications in a language other than English	1	Articles not available in English
No full text	3	Full versions of articles not available (e.g., only abstract)
Total Excluded	10	Exclusions at the screening stage + full-text

**Table 3:** Summary of OS/PFS/QoL/AE for clinical trials.

Research	Type of Intervention	OS	PFS	QoL	AE/Security
Amaral et al., 2025 [10]	KD 3:1 + RT/TMZ	Median OS ambiguous; A signal of potential improvement	Not reported	Stable QoL (EORTC QLQ-C30/BN20)	No heavy AEs; Good tolerance
McGill M. et al., 2020 [16]	KD (feasibility)	Not reported	Not reported	Stable in dieters; decrease in QoL in those who quit	AE mild; dietary difficulties
Voss et al., 2022 [17]	KD-IF + re-irradiation	OS ambiguous; low glucose levels as a marker of better prognosis	Not reported	Not rated	Good tolerance; no heavy AE
Sargaço et al., 2022 [18] (systematic review—Primary data were not double-counted)	Different protocols KD	OS 12–22 months (based on primary research)	Variable; protocol dependent	Dependent on the study	AE mild; KD safe
Montella et al., 2021 [19] (narrative review—data were not counted as primary)	Different protocols KD	OS ambiguous	Not reported	Not reported	KD Safe, difficult to maintain

**Table 4:** Characteristics and findings of studies included in this review.

Author, Year	Country	Aim of the Study	Study Design	Participants	Results and Findings
Duraj T. et al., 2024 [20]	USA	To propose a clinical research framework for ketogenic metabolic therapy (KMT) in glioblastoma multiforme	Conceptual framework/study protocol	Adult Patients with Glioblastoma Multiforme (GB)—Conceptual Paper/Study Protocol Proposal	✓ KMT may be the basis for a new therapeutic approach. ✓ The most important biomarker of glioblastoma treatment efficacy is GKI (glucose-ketone index). ✓ Diet alone is not enough—simultaneous blocking of glycolysis and glutaminolysis is necessary. ✓ KMT should be considered a “metabolic preparation” before other therapies. ✓ The most important biomarker of efficacy is GKI (glucose-ketone index). ✓ KMT is a promising, low-toxicity strategy that should be included in clinical trials as part of a combination therapy.
Valerio J. et al., 2024 [7]	USA	Conduct a systematic review of clinical trials to evaluate the role of the KD (KD) in the treatment of glioblastomas, especially glioblastoma multiforme (GB), summarize the mechanisms of action of KD, evaluate the safety, feasibility and impact of KD on patient survival, collect and analyze all available clinical trials in which KD has been used alone or as an adjunct therapy	Systematic review	-	✓ KD is safe and well-tolerated in most individuals who participated in clinical trials. ✓ Although the efficacy of this treatment in humans is still uncertain, clinical trials have shown a beneficial effect. ✓ Preclinical evidence is more consistent and indicates that using this treatment as an adjunct treatment for glioblastomas, particularly in GB reports and clinical trials, may have a beneficial effect.
Montella L. et al., 2021 [19]	Italy	to provide an overview of current knowledge on the role of the KD (KD) in the treatment of glioblastoma multiforme (GB))	Narrative review	-	✓ There are strong mechanistic reasons to consider KD as potentially beneficial in GB. ✓ KD may theoretically weaken GB cells, which are glucose-dependent. ✓ KD is not unequivocally effective—studies have conflicting results. ✓ KD is difficult to maintain and requires close monitoring. ✓ KD can be a valuable adjunct therapy, but it does not replace oncological treatment. ✓ KD has a solid biological basis and some promising data, but its effectiveness in GB is equivocal, dependent on the metabolic context, and requires significantly better clinical trials.
Dal Bello S. et al., 2022 [11]	Italy	summary of current scientific knowledge on the use of the KD (KD) as an adjunct therapy in the treatment of glioblastomas and glioblastoma multiformes (glioblastoma)	Narrative review	-	✓ KD may have anti-cancer effects at multiple levels ✓ KD may reduce peritumoral edema and steroid requirements ✓ KD is not a standalone anti-cancer therapy ✓ The KD has a solid biological basis and promising results in preclinical studies, but large clinical trials are lacking to clearly confirm its effectiveness in the treatment of glioblastomas and GB
Sargaço B. et al., 2022 [18]	Portugal	The main objective of the present systematic review was to analyze and systematize the clinical studies that tested KD in the context of glioblastoma treatments	Systematic review	-	✓ The KD may prolong the survival of patients with glioblastomas ✓ The variety of KD protocols makes it difficult to compare results ✓ Better, larger studies are needed to confirm the effectiveness of KD as an adjunctive therapy.
Amaral LJ. et al., 2025 [10]	USA	Safety assessment of a 16-week KD (3:1) used concurrently with standard treatment (radiotherapy + temozolomide) in patients with newly diagnosed glioblastoma multiforme (GB).	Interventional clinical study (safety/feasibility)	adults > 18 years of age, with newly or recently diagnosed GB, within 3 months of diagnosis, in good general condition (KPS ≥ 70), with BMI ≥ 22 kg/m^2^	✓ The KD is safe for GB patients ✓ The KD, combined with standard GB treatment, is feasible, maintains high ketosis, does not worsen quality of life, and may improve survival outcomes, although this cannot be confirmed without controlled studies.
McKerill E. et al., 2025 [21]	UK	understanding the scope of research on (KMT) as an adjunct treatment to standard therapy	Scoping review	-	✓ No firm conclusions can be drawn regarding the efficacy of KMT in the treatment of glioblastoma multiforme. ✓ The scope of research on KMT is limited, with few completed clinical trials compared to other treatment options for glioblastoma multiforme.
Winter SF. et al., 2017 [22]	Germany	review of the scientific basis and available data on the use of KMT in the treatment of patients with malignant glioblastoma	Narrative review	-	✓ a growing number of ongoing clinical trials suggest that KMT is emerging as a potential therapeutic option and may be combined with existing anticancer treatments for malignant glioblastoma
McGill M. KJ. et al., 2018 [23]	UK	a review of the evidence on the effectiveness and acceptability of different ketogenic (KD) diets in the treatment of glioblastoma	Narrative review	-	✓ The effectiveness and acceptability of KD in the treatment of glioblastomas is unknown. ✓ High-quality randomized controlled trials are necessary.
McGill M. KJ. et al., 2020 [16]	UK	the use of KDs (KDs) as an adjuvant therapy for patients with glioblastoma (GB)	Feasibility clinical study	Twelve newly diagnosed patients with GB	✓ A KD as an adjunctive therapy for GB is feasible but difficult to maintain. ✓ The KD is difficult to accept and burdens patients. ✓ Caregiver support is crucial. ✓ Quality of life decreased in those who dropped out but remained stable in those who persisted. ✓ A KD is feasible in clinical settings but requires a shorter duration. ✓ The study did not evaluate the effectiveness of the KD—only its feasibility.
Voss M. et al., 2022 [17]	Germany	to investigate the effect of a calorie-restricted KD and intermittent fasting (KD-IF) on re-radiation therapy for relapsed brain tumors	Interventional clinical study (KD-IF + re-irradiation)	50 patients	✓ The study’s strict calorie goals were well tolerated by patients with recurrent brain cancer. ✓ The short diet plan led to significant metabolic changes, and low glucose levels proved to be a potential marker for a better prognosis.
Noorlag L. et al., 2019 [24]	Netherlands	summary of preclinical and clinical data on KCRD in glioblastomas	Narrative review	-	✓ KCRD has shown a positive effect on malignant glioblastomas in published preclinical studies. ✓ Preliminary clinical data suggest that KCRD is safe and feasible. ✓ The effectiveness of KCRD in improving the survival and quality of life of glioblastoma patients remains to be confirmed in prospective studies.

**Table 5:** Breakdown of in vitro/in vivo studies included in the review.

Type of Study	Research Model	Publication	Characteristics
In vivo (animals)	Rat model	Champ et al., 2014 [25]	KD as a supportive therapy; Effects on tumor growth and metabolic markers.
In vivo (clinic)	Patients with GB	McGill M. et al., 2020 [16]	Feasibility KD; tolerance, quality of life, metabolic parameters.
In vivo (clinic)	Patients with GB	Amaral et al., 2025 [10]	KD 3:1 + RT/TMZ; OS, PFS, QoL, safety.
In vivo (clinic)	Relapse patients GB	Voss et al., 2022 [17]	KD-IF + re-irradiation; metabolic parameters, tolerance.
In vivo (clinic)	Patients with GB	Sargaço et al., 2022 [18] (review—Raw data not double-counted)	Summary of clinical trial results; OS/PFS from primary studies

**Table 6:** Components of the KD and their effects on glioblastoma [24,33].

Diet Ingredient/Element	Mechanism of Metabolic Action	Effects on Glioblastoma Cells	Therapeutic Effect/Potential Clinical Impact
Fats (MCT oils, saturated and unsaturated fats)	They increase the production of ketone bodies (βhydroxybutyrate, acetone, acetoacetate); limit glucose as an energy source	Glioblastoma cells cannot use ketones effectively; there is an energy deficit	Slowing tumor growth, increasing sensitivity to radiation therapy and chemotherapy
Protein (moderate supply)	Maintains muscle mass without stimulating gluconeogenesis; limits the availability of amino acids for cancer cells	Reduces the use of glutamine and other amino acids by glioblastoma cells	Limiting tumor cell proliferation and tumor metabolic stress
Ketone bodies (β-hydroxybutyrate, acetoacetate)	An alternative source of energy for neurons; modulate gene expression and oxidative stress	They induce oxidative stress in glioblastoma cells that have impaired mitochondria	Increase apoptosis of cancer cells, stabilization of neuronal function
Low carbohydrate supply	Lowers glucose and insulin levels; inhibits PI3K/Akt/mTOR signaling pathways	Glioblastoma loses its main source of energy; inhibition of proliferation and angiogenesis	Reduction of tumour growth rate, reduction of blood vessel formation
Omega3 fatty acids (e.g., from fish, flaxseed)	Anti-inflammatory, stabilization of cell membranes	Inhibit NFκB activation and production of pro-inflammatory cytokines	Reduction of inflammation in the tumor microenvironment, improvement of immune response
MCTs (medium-chain triglycerides)	Rapidly metabolized into ketones; support the maintenance of ketosis	Helps you achieve stable ketone levels without overloading your fats	Maintaining stable ketosis, improving diet tolerance and patients’ quality of life
Polyphenols and antioxidants (e.g., from avocados, olive oils)	Neutralize free radicals, support mitochondrial functions	They protect healthy cells from oxidative stress, intensify stress in tumor cells	Increasing the selectivity of the diet—protecting neurons, damaging cancer cells

**Table 7:** A synthetic summary of the results of the reviews.

ClinicalTrials.gov ID	Status
NCT03075514	Completed
NCT00575146	Completed
NCT01865162	Completed
NCT02939378	Unknown status
NCT04691960	Active, not recruiting
NCT02302235	Completed
NCT05183204	Suspended
NCT05708352	Recruiting
NCT02046187	Terminated
NCT03278249	Completed
NCT03451799	Completed
NCT01754350	Completed
NCT02286167	Completed
NCT04730869	Completed
NCT01535911	Active, not recruiting

## Data Availability

No new data were created or analyzed in this study. Data sharing is not applicable to this article.

## Abbreviations

The following abbreviations are used in this manuscript:

GB	Glioblastoma multiforme
KD	KD
PCC	Population–Concept–Context
MCT-KD	Medium-chain triglyceride KD
PRISMA-ScR	Preferred Reporting Items for Systematic reviews and Meta-Analyses extension for Scoping Reviews
KMT	KMT
GKI	glucose-ketone index
KD-IF	KD and intermittent fasting
KCRD	Ketogenic or Caloric Restricted Diets
MCTs	medium-chain triglycerides
